# Plasma concentrations and intakes of amino acids in male meat-eaters, fish-eaters, vegetarians and vegans: a cross-sectional analysis in the EPIC-Oxford cohort

**DOI:** 10.1038/ejcn.2015.144

**Published:** 2015-09-23

**Authors:** J A Schmidt, S Rinaldi, A Scalbert, P Ferrari, D Achaintre, M J Gunter, P N Appleby, T J Key, R C Travis

**Affiliations:** 1 Cancer Epidemiology Unit, Nuffield Department of Population Health, University of Oxford, Oxford, UK; 2 International Agency for Research on Cancer, Lyon, France; 3 Department of Epidemiology and Biostatistics, School of Public Health, Imperial College London, London, UK

## Abstract

**Background/Objectives:**

We aimed to investigate the differences in plasma concentrations and in intakes of amino acids between male meat-eaters, fish-eaters, vegetarians and vegans in the Oxford arm of the European Prospective Investigation into Cancer and Nutrition.

**Subjects/Methods:**

This cross-sectional analysis included 392 men, aged 30–49 years. Plasma amino acid concentrations were measured with a targeted metabolomic approach using mass spectrometry, and dietary intake was assessed using a food frequency questionnaire. Differences between diet groups in mean plasma concentrations and intakes of amino acids were examined using analysis of variance, controlling for potential confounding factors and multiple testing.

**Results:**

In plasma, concentrations of 6 out of 21 amino acids varied significantly by diet group, with differences of −13% to +16% between meat-eaters and vegans. Concentrations of methionine, tryptophan and tyrosine were highest in fish-eaters and vegetarians, followed by meat-eaters, and lowest in vegans. A broadly similar pattern was seen for lysine, whereas alanine concentration was highest in fish-eaters and lowest in meat-eaters. For glycine, vegans had the highest concentration and meat-eaters the lowest. Intakes of all 18 dietary amino acids differed by diet group; for the majority of these, intake was highest in meat-eaters followed by fish-eaters, then vegetarians and lowest in vegans (up to 47% lower than in meat-eaters).

**Conclusions:**

Men belonging to different habitual diet groups have significantly different plasma concentrations of lysine, methionine, tryptophan, alanine, glycine and tyrosine. However, the differences in plasma concentrations were less marked than and did not necessarily mirror those seen for amino acid intakes.

## Introduction

Amino acids are the building blocks of proteins^1^ and are additionally utilised as a source of energy. They are necessary for the synthesis of a wide variety of compounds, including neurotransmitters, haem and DNA. Humans need daily supplies of protein including adequate amounts of essential amino acids, which cannot be synthesised endogenously.^2^ The amino acid composition of animal protein better resembles the body's need than that of single sources of plant protein. However, as different plant proteins complement each other in terms of amino acid composition, diets without animal products can fulfil the requirement.

Although circulating concentrations of amino acids are subject to homoeostatic control,^3^ they are also affected by diet, metabolism, lifestyle and genetic factors.^4^ Individuals who exclude some or all animal products from their diet may thus have different circulating concentrations compared with those of meat-eaters, because of differences in dietary protein sources, lifestyle and metabolism. Previous studies have found some variations in circulating concentrations or intakes of amino acids between vegans, vegetarians and omnivores.^5, 6, 7, 8, 9, 10^ However, none of these studies have investigated a wide range of amino acids in terms of both concentration and dietary intake, and limited numbers of participants were included (*n*<75).

The aim of this study was therefore to investigate the differences in plasma concentrations and in dietary intakes of amino acids between male meat-eaters, fish-eaters, vegetarians and vegans in the Oxford arm of the European Prospective Investigation into Cancer and Nutrition (EPIC-Oxford).

## Subjects and methods

### Study population

The EPIC-Oxford cohort comprises 65 000 participants including 14 600 men over the age of 20 years recruited from throughout the United Kingdom in 1993–2001. The study has been described in detail elsewhere.^11^ The primary aim of this cohort is to investigate diet, lifestyle and risk of cancer and other chronic diseases in people with a wide range of different dietary habits. Therefore, recruitment was aimed at vegetarians and vegans, as well as the general population. Most participants (89%) were recruited by post, whereas the remaining participants were recruited via general practitioners' surgeries. All participants gave written informed consent, and the protocol for EPIC-Oxford was approved by a multicentre research ethics committee (MREC/02/0/90).

The following eligibility criteria for the current analysis were applied to limit variation in amino acid concentrations and intakes because of factors other than diet group: male sex; age 30–49 years; provision of a blood sample at recruitment; known smoking status and diet group; response to 80% or more of the relevant questions in the food frequency questionnaire (FFQ) and a daily energy intake between 3.3 and 16.7 MJ (800–4000 kcal); and no prior cancer (excluding non-melanoma skin cancer), cardiovascular disease or treatment for any long-term illness or condition at recruitment. Of the 110 eligible vegans (who do not eat any animal products), we selected all those aged 30–39 years and randomly selected four out of every five aged 40–49 years. In addition, eligible meat-eaters (who eat meat), fish-eaters (who do not eat meat but do eat fish) and vegetarians (who do not eat meat or fish but do eat dairy products and/or eggs) were randomly selected in equal numbers within strata of the ages 30–39 and 40–49 years. A total of 392 men, 98 in each diet group, were included in this analysis.

### Blood sampling and laboratory analysis

At recruitment, participants had a blood sample taken at their local general practitioner's surgery. Fasting was not required, but time since last food or drink was recorded. Whole blood was sent at ambient temperature to a laboratory in Norfolk, centrifuged and aliquoted into 0.5 ml straws and stored in liquid nitrogen (−196 °C) until 2011 and subsequently in electric freezers (−80 °C).

In 2013, samples were transported on dry ice to the International Agency for Research on Cancer, Lyon, France, for assay. Before the assay for this study, samples had undergone two or three thaw–freeze cycles; samples from each diet group were equally distributed by the number of cycles. The targeted metabolomics assay, BIOCRATES Absolute*IDQ* p180 Kit (Biocrates, Innsbruck, Austria), quantified 21 amino acids using liquid chromatography-tandem mass spectrometry. For quality control, four to six blinded samples of pooled plasma were included in each batch. The laboratory technicians were blinded to the diet group and the quality control samples. The median (range) overall coefficient of variation for the amino acids was 11.8% (5.4–17.3). No samples had concentrations below the lower limit of quantification, whereas for five amino acids a few values (*n*⩽2) were above the highest standard and were set equal to this value (400 μmol/l for leucine, histidine, threonine and ornithine, and 800 μmol/l for glutamate). For some participants, it was not possible to assess the concentration of lysine (*n*=11), phenylalanine (*n*=93), tryptophan (*n*=2), glutamine (*n*=2), proline (*n*=45) or ornithine (*n*=1) accurately because of signal saturation of the detector in the mass spectrometer; thus, no data were available. The number of unavailable concentrations did not differ significantly by diet group for lysine, phenylalanine or proline (no comparison was made for the other amino acids because of the low number of affected samples). Participants were excluded only from analyses of amino acids for which they did not have data.

### Diet and lifestyle

At recruitment, participants completed a validated semi-quantitative FFQ,^12, 13^ with additional questions on smoking status, height and weight. There was good agreement between self-reported and measured height and weight from a sub-sample of the cohort (correlation coefficient (*r*)>0.9).^14^ If available, measured values were used.

Categorisation of the four diet groups was based on the answers to the questions 'Do you eat any meat; fish; dairy products; and eggs?' Participants were further asked how often they consumed 130 or 113 foods and drinks, depending on their diet group. Mean daily food and drink intakes were estimated using specified portion sizes,^15^ and mean daily nutrient intakes were estimated mostly using data from the fifth edition of ‘McCance and Widdowson's The Composition of Foods' and its supplements.^16, 17, 18, 19, 20, 21, 22, 23, 24, 25^

To estimate intakes of 18 amino acids, data from the United States Department of Agriculture, National Nutrient Database for Standard Reference, release 26,^26^ were used, because such data are only available for a limited number of foods in the United Kingdom. All UK foods were matched to the nearest food in the US nutrient database, paying specific attention to match on the total protein content so that scaling was not necessary. The data in the US database are based on high-performance liquid chromatography following protein hydrolysis and thus mainly represent protein amino acids. The US and UK nutrient tables do not include information on asparagine or glutamine because of the assay methods used:^27^ acid hydrolysis transforms asparagine and glutamine to aspartate and glutamate, respectively, which are consequently overestimated in nutrient tables.

To estimate protein intakes from different sources, all items in the FFQ were categorised in seven distinct food types, that is, non-soya plant, soya, meat, fish, dairy, egg and mixed animal products, based on the main source of protein in the food.

### Statistical analysis

Participant characteristics, nutrient intakes and factors related to blood handling were compared across diet groups.

All amino acid concentrations and intakes were logarithmically transformed to approximate the normal distribution, except concentrations of tryptophan, aspartate and glutamine, which were closer to the normal distribution when on the original scale.

Differences between diet groups in mean concentrations and intakes of amino acids were tested using analysis of variance. The analyses of concentrations and intakes were both adjusted for age (30–34; 35–39; 40–44; 45–49 years), body mass index (BMI; <22.5; 22.5–24.9; ⩾25 kg/m^2^; unknown), smoking status (never; former; current) and alcohol intake (<1; 1–7; 8–15; ⩾16 g/d). Time since last food or drink at blood collection (<1.5; 1.5–2.9; 3.0–4.4; ⩾4.5 h; unknown) and time between blood collection and processing (fourths of the distribution; unknown) were included in the model for plasma concentrations only, whereas energy intake (continuous) was included in the model for intakes. A sensitivity analysis further adjusting for physical activity (inactive; low activity; moderately active; very active; unknown)^28, 29^ was conducted for both plasma concentrations and intakes of amino acids.

Furthermore, Spearman's rank correlation coefficients were calculated between individual plasma amino acids, between plasma amino acids and percentage energy from protein from food types (excluding diet groups which do not consume the food type) and between amino acids measured in plasma and diet.

Conventional two-sided *P*-values are shown, but all results have been interpreted after allowance for multiple testing using the Bonferroni method; the per-test significance level was 0.05 divided by the number of tests.

All analyses were performed using Stata Statistical Package version 13.1 (Stata Corp., College Station, TX, USA).

## Results

Fish-eaters, vegetarians and vegans had on average followed their diet for 11, 12 and 9 years, respectively.

BMI differed by up to 2.3 kg/m^2^ between diet groups; it was highest in meat-eaters and lowest in vegans (Table 1). Similarly, energy intake and percent energy from protein were highest in meat-eaters and lowest in vegans. Separating the protein intake by food types revealed differences in percent energy from protein from all plant products, non-soya and soya plant products; meat-eaters had the lowest intake, whereas vegans consumed the highest amount. Protein from all animal products also differed by diet group; it was highest in meat-eaters and lowest in vegetarians (zero in vegans by definition). However, no differences between diet groups were observed for the four types of animal products where comparison was possible (fish, dairy, egg and mixed animal products). There was some variation by diet group in median duration since last meal or drink at blood collection (up to 30 min) and in time between blood collection and processing (up to 19 h), although after adjustment for multiple testing this was not statistically significant (Supplementary Table 1).

The concentrations of 6 of the 21 plasma amino acids varied significantly by diet group after allowing for multiple testing (Table 2). For lysine, methionine, tryptophan and tyrosine fish-eaters and vegetarians generally had the highest concentrations, followed by meat-eaters, whereas vegans had the lowest concentrations for all four. For alanine, the highest concentration was seen in fish-eaters, followed by vegetarians and vegans, and lowest in meat-eaters. The largest difference was seen for glycine; mean concentration in vegans was 16% higher than in meat-eaters, who had the lowest concentration, with intermediate concentrations for fish-eaters and vegetarians. Differences between diet groups in concentrations of leucine, valine, histidine, aspartate, glutamate, glutamine, citrulline and ornithine were not statistically significant after correction for multiple testing. Further adjusting for physical activity did not materially change the findings (results not shown).

**Figure 1:**
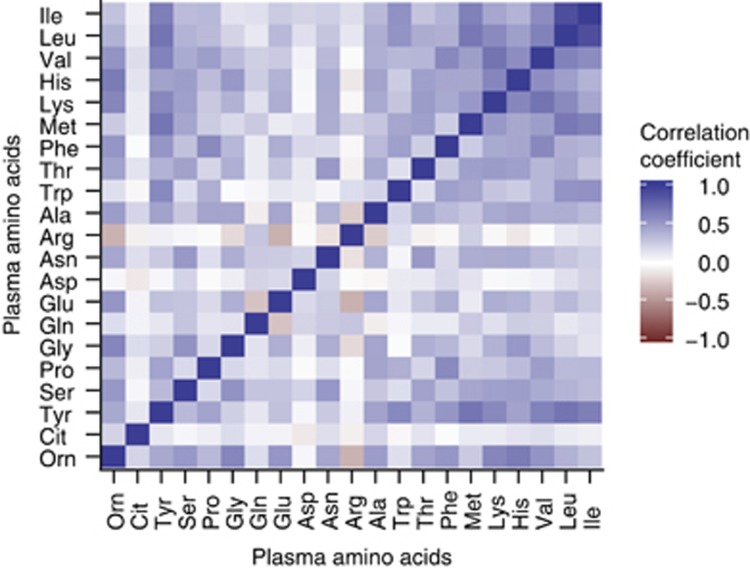
Correlation matrix for plasma amino acid concentrations. Spearman's rank correlation coefficients between plasma amino acid concentrations. The colours indicate the strength and direction of the correlations. Ala, alanine; Arg, arginine; Asn, asparagine; Asp, aspartate; Cit, citrulline; Gln, glutamine; Glu, glutamate; Gly, glycine; His, histidine; Ile, isoleucine; Leu, leucine; Lys, lysine; Met, methionine; Orn, ornithine; Phe, phenylalanine; Pro, proline; Ser, serine; Thr, threonine; Trp, tryptophan; Tyr, tyrosine; Val, valine.

Concentrations of many of the plasma amino acids were correlated (Figure 1); 62% of the correlations were statistically significant after allowance for multiple testing (–0.23⩾*r*⩾0.23; *P*<0.0002). The strongest positive correlations were observed between the essential amino acids (*r*⩽0.91). The essential amino acids were also positively correlated with several other amino acids, especially strongly with tyrosine. For all amino acids, the vast majority of correlations were positive, but some negative correlations were found.

Estimated mean intakes of all 18 amino acids varied by diet group (*P*<0.0001 for all; Table 3). Meat-eaters had the highest intakes, followed by fish-eaters, vegetarians and vegans, except for arginine, aspartate, glycine and proline. For these amino acids, meat-eaters also had the highest intakes, but were followed by vegans for arginine and glycine, whereas other deviations from the above pattern were seen for aspartate and proline. The largest percentage differences between diet groups were for intakes of lysine and methionine, which in vegans were 56% and 53% of those in meat-eaters, respectively. Including physical activity in the statistical model did not materially change the findings (results not shown).

Intakes and plasma concentrations of amino acids were not strongly correlated (Table 4). However, significant positive correlations were found for leucine, lysine, methionine, tryptophan and tyrosine (*r*⩽0.21), after allowing for multiple testing.

Concentrations of some plasma amino acids were correlated with intake of protein from plant products, after allowing for multiple testing (Supplementary Table 2). For soya products, inverse correlations were seen with valine, lysine, methionine and tyrosine. A similar pattern was seen for non-soya plant products, although only correlations for lysine and tyrosine were significant. In contrast, glycine was positively correlated with protein from soya products. There were no statistically significant correlations between plasma amino acids and protein from animal food types, after allowing for multiple testing. However, positive correlations of similar magnitude to the ones for plant foods were seen for dairy and egg products, although these analyses had lower power because vegans did not contribute.

## Discussion

This analysis showed that plasma concentrations of six amino acids, namely lysine, methionine, tryptophan, alanine, glycine and tyrosine, differed by habitual diet group. These differences were less pronounced than, and did not mirror those observed for amino acid intakes (the majority of which were highest in meat-eaters followed by fish-eaters, then vegetarians and finally vegans). Concentrations of the essential amino acids and tyrosine were generally highest in fish-eaters and vegetarians, followed by meat-eaters and all lowest in vegans. Observing the lowest concentrations in vegans was not unexpected, as essential amino acids are not synthesised endogenously and are less abundant in plant than animal protein.^1, 2^ For alanine, the highest concentrations were observed in fish-eaters, followed by vegetarians and vegans, and were lowest in meat-eaters. Vegans had the highest concentration of glycine followed by fish-eaters and vegetarians, whereas meat-eaters had the lowest concentrations. Whereas most plasma amino acids were correlated with each other, only a few significant correlations were seen between plasma concentrations and dietary intakes of amino acids and between plasma amino acids and intakes of protein from different food sources.

The observed differences between diet groups in amino acid intakes may be explained by the protein intake being lower the more animal products were excluded from the diet. The observed trend in protein intake may also partly reflect an increasing degree of underestimation of intake as more animal foods are omitted; some plant foods eaten by vegetarians and vegans may not be in the FFQ, and vegetarians and vegans may eat larger portions of some plant foods than the standard portion sizes assigned. Also, the validation of the FFQ showed that protein intake was particularly difficult to estimate.^12, 13^

Lysine or the sum of methionine and cysteine are the limiting amino acids in many plant proteins,^2^ meaning that they are present in the lowest amount relative to the requirement. This is in line with our findings of lysine and methionine intakes accounting for the largest differences between vegans and meat-eaters (nearly 50% lower in vegans).

Our results on amino acid intakes are further supported by a previous study that found similar percent differences in intakes of lysine and methionine between omnivores, lacto-ovo-vegetarians and vegans^10^ to those in the current study. Somewhat contrasting, another study found that intakes of several amino acids, including lysine, were highest in lactovegetarians, followed by meat-eaters and lowest in vegans, whereas vegans had the highest intake of other amino acids, including the combination of methionine and cysteine.^5, 6^ However, in this latter study, the number of participants was small (*n*=12 men), and no statistical tests were performed.

The plasma concentration of amino acids is a result of a complex interplay between dietary intake, tissue breakdown and *de-novo* synthesis^3^ and other factors such as gut microbial synthesis increasing the concentration^30^ and tissue uptake and excretion reducing the concentration.^3^ This, in addition to the recognised limitations of estimating nutrient intakes using a FFQ,^31^ might at least partly explain the differences in our results for plasma and dietary amino acids by diet group, as well as the limited strength of correlation between plasma concentrations and intakes. Furthermore, as some diet groups were excluded from the analysis of amino acid concentrations and intakes of protein from animal sources because of zero intake, lower power has also contributed to the lack of significant correlations in this analysis.

The largest percentage difference between meat-eaters and vegans in plasma concentration was found for glycine (16%), for which vegans had the highest concentration. Plasma glycine concentration was also positively correlated with intake of protein from plant products––especially soya products, which have high glycine content^26^ and are consumed in greater quantities by the vegans than the other groups. This could at least in part explain why vegans had the highest glycine concentration, although differences in metabolism between diet groups could also contribute to the finding.

There are a few previous studies available on plasma amino acid concentrations in individuals who exclude some or all animal products from their diet. The differences by diet group we observed for lysine and tyrosine were supported by similar findings from previous studies, as were the null results for isoleucine, phenylalanine, asparagine, proline, serine and ornithine.^7, 9^ For leucine, valine, histidine, threonine, arginine and citrulline an association was found in previous studies but not in ours, whereas the opposite was the case for methionine, tryptophan, alanine and glycine.^7, 8, 9^ However, the previous studies were all limited by small numbers of participants (*n*<36).

The results of the current study have to be interpreted in the light of its strengths and limitations. To our knowledge, this is the largest study to date of amino acids in the circulation or in the diet by habitual diet group, and on average participants had followed their diet for several years. With 98 participants in each diet group, the study had more than 80% power to detect a 10% difference in mean amino acid concentration (for example lysine) between diet groups. Moreover, data were available on both plasma concentrations and dietary intakes of amino acids, as well as on a wide range of covariates, for example BMI, which is related to amino acid concentrations.^32^ These strengths facilitated robust comparisons between diet groups while limiting the likelihood of confounding, and made it possible to examine the correlations between dietary intake and circulating concentration of amino acids. The main limitations are related to the measurement of amino acid concentration and intake. Firstly, the measurements of plasma amino acids could be affected by pre-analytic conditions such as thaw–freeze cycles. However, most amino acids have been found to be stable during two thaw–freeze cycles,^33^ and in the current study, the number of thaw–freeze cycles was independent of diet group. Other pre-analytical conditions, which we do not have information about, are most likely also not related to diet group. Pre-analytical factors are therefore unlikely to explain our findings. Secondly, although care was taken to match the food items in the FFQ with appropriate foods in the US database, the amino acid content of the selected foods may differ somewhat between the United States and the United Kingdom.

It is not known whether the observed differences by diet group in plasma concentrations or dietary intakes of amino acids might translate to differences in health and disease status. One study of type 2 diabetes found serum concentrations of glycine to be associated with a decreased risk and phenylalanine to be associated with an increased risk of the disease,^34^ whereas a second study reported a higher risk of diabetes in individuals with higher plasma concentrations of isoleucine, leucine, valine, tyrosine and phenylalanine.^35^ Associations have also been found between high intake of methionine and lower risk of breast^36^ and pancreatic cancers.^37^ Finally, essential amino acids, especially leucine, are important for muscle protein synthesis and may contribute to maintaining skeletal muscle mass in elderly people and after exercise.^38^ Future studies might help develop our understanding of the interplay between diet, alterations in amino acid metabolism and health and disease status.

In conclusion, British men belonging to different habitual diet groups have significantly different plasma concentrations of lysine, methionine, tryptophan, alanine, glycine and tyrosine. The differences in plasma concentrations were less marked than and did not closely mirror those seen for amino acid intakes, which were all highest in meat-eaters and mostly lowest in vegans.

## Figures and Tables

**Table 1:** Characteristics and intakes of nutrients by habitual diet group

	*Median (inter-quartile range) or* n *(%)^a^*				P_*difference^b^*_
	*Meat-eaters (*n=*98)*	*Fish-eaters (*n=*98)*	*Vegetarians (*n=*98)*	*Vegans (*n=*98)*	
*Characteristics*					
Age at blood collection, years	44 (37, 44)	41 (36, 45)	43 (36, 44)	40 (35, 44)	>0.9
BMI^c^, kg/m^2^	24.4 (22.1, 25.9)	22.7 (21.2, 24.2)	22.7 (21.5, 24.9)	22.1 (20.4, 24.0)	**0.0001**
Current smoking, *n* (%)	14 (14.3)	9 (9.2)	7 (7.1)	7 (7.1)	0.1
Very physically active, *n* (%)	26 (28.0)	17 (17.9)	12 (13.2)	31 (33.7)	0.02
					
*Nutrient intake*					
Energy, kJ	9137 (7921, 10957)	8734 (7511, 10127)	9011 (7597, 10933)	7652 (6091, 8932)	**0.0001**
Protein, % of energy	15.0 (13.6, 16.9)	13.6 (12.3, 15.4)	13.3 (11.8, 14.2)	12.6 (11.6, 13.9)	**0.0001**
Protein from all plant products,^d^ % of energy	6.0 (5.4, 7.1)	7.4 (6.5, 8.8)	7.8 (6.8, 9.3)	12.3 (11.3, 13.7)	**0.0001**
Protein from non-soya plant products,^d^ % of energy	5.9 (5.2, 6.9)	7.0 (6.2, 7.9)	7.2 (6.4, 8.3)	9.6 (8.6, 10.7)	**0.0001**
Protein from soya products,^d^ % of energy	0.0 (0.0, 0.0)	0.3 (0.2, 1.0)	0.4 (0.2, 1.1)	2.8 (1.8, 3.4)	**0.0001**
Protein from all animal products,^d^ % of energy	8.4 (6.7, 10.5)	5.6 (4.7, 7.5)	4.6 (3.8, 6.0)	−	**0.0001**
Protein from meat products,^d^ % of energy	3.3 (1.6, 4.7)	−	−	−	−
Protein from fish products,^d^ % of energy	1.0 (0.6, 1.6)	1.2 (0.6, 1.7)	−	−	0.5
Protein from dairy products,^d^ % of energy	3.4 (2.4, 4.3)	3.6 (2.5, 4.7)	3.7 (2.9, 4.9)	−	0.1
Protein from egg products,^d^ % of energy	0.2 (0.1, 0.3)	0.2 (0.1, 0.5)	0.2 (0.1, 0.4)	−	0.1
Protein from mixed animal products,^d^ % of energy	0.5 (0.2, 0.7)	0.5 (0.4, 0.8)	0.5 (0.4, 0.8)	−	0.02
Carbohydrates, % of energy	51.2 (47.1, 55.6)	52.2 (48.3, 57.4)	54.3 (48.7, 58.2)	55.6 (52.1, 60.9)	**0.0001**
Fat, % of energy	31.9 (28.9, 34.9)	32.2 (27.2, 35.0)	31.3 (27.4, 35.3)	30.4 (25.2, 34.4)	0.2
Alcohol, g/d	9.8 (2.5, 17.2)	9.7 (2.9, 29.7)	10.2 (5.1, 23.0)	5.1 (1.0, 14.0)	**0.0007**

Abbreviation: BMI, body mass index.

a Values are median (inter-quartile range) if not otherwise specified as *n* (%).

b Differences between diet groups were tested using the Kruskal–Wallis analysis of variance and the *χ*^2^ test for continuous and categorical variables, respectively. Conventional *P*-values are shown and those marked in bold were significant after Bonferroni correction (*P*<0.0023; not all tests performed are shown).

c BMI was unknown for 20 participants.

d Foods were categorised into food types based on the main protein source of the food. Medians and inter-quartile ranges do not necessarily add up because mixed foods for which there are non-vegetarian as well as vegetarian and vegan versions were categorised as one food type only, for example, mixed animal products.

**Table 2:** Plasma concentrations of amino acids by habitual diet group^a^

	*Geometric mean concentration (95% confidence interval), μmol/l % mean difference compared with meat-eaters*				P_*difference*_^b^
	*Meat-eaters*	*Fish-eaters*	*Vegetarians*	*Vegans*	
	(n=*98)*	(n=*98)*	(n=*98)*	(n=*98)*	
					
*Branched-chain essential amino acids*					
Isoleucine	99 (94, 103)	99 (95, 103)	102 (97, 106)	96 (92, 100)	0.3
	**Ref.**	**0**	**+3**	**−3**	
Leucine	205 (197, 214)	208 (200, 216)	210 (202, 218)	191 (184, 199)	0.005
	**Ref.**	**+1**	**+2**	**−7**	
Valine	230 (221, 239)	233 (225, 242)	233 (225, 242)	217 (209, 225)	0.02
	**Ref.**	**+1**	**+1**	**−6**	
*Other essential amino acids*					
Histidine	114 (110, 118)	122 (118, 126)	119 (115, 122)	117 (113, 120)	0.04
	**Ref.**	**+7**	**+4**	**+2**	
Lysine^c^	241 (230, 253)	242 (232, 253)	234 (224, 245)	210 (201, 219)	**<0.0001**
	**Ref.**	**+1**	**−3**	**−13**	
Methionine	29 (28, 31)	30 (29, 31)	31 (30, 32)	27 (26, 28)	**0.0001**
	**Ref.**	**+2**	**+5**	**−8**	
Phenylalanine^c^	95 (92, 99)	101 (97, 105)	100 (97, 104)	97 (93, 101)	0.1
	**Ref.**	**+6**	**+5**	**+2**	
Threonine	164 (158, 170)	168 (163, 174)	167 (162, 173)	165 (159, 171)	0.7
	**Ref.**	**+3**	**+2**	**+1**	
Tryptophanc,^d^	69 (67, 72)	71 (68, 73)	72 (70, 74)	65 (63, 68)	**0.001**
	**Ref.**	**+2**	**+4**	**−6**	
					
*Non-essential amino acids*					
Alanine	564 (540, 590)	644 (618, 671)	627 (602, 654)	621 (595, 648)	**0.0004**
	**Ref.**	**+14**	**+11**	**+10**	
Arginine	51 (46, 57)	44 (40, 49)	44 (40, 49)	44 (39, 48)	0.1
	**Ref.**	**−14**	**−14**	**−15**	
Asparagine	92 (89, 95)	97 (94, 101)	96 (92, 99)	98 (95, 102)	0.07
	**Ref.**	**+6**	**+4**	**+7**	
Aspartate^d^	66 (62, 69)	64 (61, 67)	69 (66, 72)	69 (66, 72)	0.04
	**Ref.**	**−3**	**+5**	**+5**	
Glutamate	261 (247, 277)	296 (281, 312)	277 (263, 292)	262 (248, 277)	0.004
	**Ref.**	**+13**	**+6**	**0**	
Glutaminec,^d^	517 (497, 536)	506 (488, 525)	525 (507, 544)	547 (529, 566)	0.02
	**Ref.**	**−2**	**+2**	**+6**	
Glycine	390 (375, 407)	422 (406, 439)	411 (395, 427)	452 (434, 470)	**<0.0001**
	**Ref.**	**+8**	**+5**	**+16**	
Proline^c^	245 (233, 258)	255 (243, 268)	265 (253, 278)	244 (233, 256)	0.06
	**Ref.**	**+4**	**+8**	**−1**	
Serine	191 (183, 198)	201 (194, 208)	197 (190, 204)	197 (190, 205)	0.3
	**Ref.**	**+5**	**+3**	**+3**	
Tyrosine	77 (74, 81)	83 (80, 86)	83 (80, 86)	73 (70, 76)	**<0.0001**
	**Ref.**	**+7**	**+7**	**−5**	
					
*Non-standard amino acids*					
Citrulline	37 (35, 39)	37 (36, 39)	41 (39, 43)	40 (38, 42)	0.02
	**Ref.**	**+1**	**+10**	**+8**	
Ornithine^c^	182 (174, 191)	195 (187, 203)	191 (184, 200)	205 (197, 215)	0.005
	**Ref.**	**+7**	**+5**	**+13**	

Abbreviation: Ref., reference.

a Adjusted for age (30–34; 35–39; 40–44; 45–49 years), body mass index (<22.5; 22.5–24.9; ⩾25 kg/m^2^; unknown), smoking status (never; former; current), alcohol intake (<1; 1–7; 8–15; ⩾16 g/d), time since last food or drink at blood collection (<1.5; 1.5–2.9; 3.0–4.4; ⩾4.5 h; unknown) and time between blood collection and processing (fourths of the distribution; unknown).

b *P*-values refer to *P* for the difference across all four diet groups calculated by analysis of variance. Conventional *P*-values are shown, and those marked in bold were significant after Bonferroni correction (*P*<0.0024).

c Missing concentration of amino acid for some participants.

d Non-transformed data were used, and arithmetic means are displayed.

**Table 3:** Intakes of amino acids by habitual diet group^a^

	*Geometric mean intake (95% confidence interval), g/d % mean difference compared with meat-eaters*				P_*difference*_^b^
	*Meat-eaters*	*Fish-eaters*	*Vegetarians*	*Vegans*	
	(n=*98)*	(n=*98)*	(n=*98)*	(n=*98)*	
*Branched-chain essential amino acids*					
Isoleucine	3.54 (3.41, 3.68)	3.12 (3.00, 3.24)	2.95 (2.85, 3.06)	2.47 (2.38, 2.57)	**<0.0001**
	**Ref.**	**−12**	**−17**	**−30**	
Leucine	6.13 (5.90, 6.35)	5.51 (5.30, 5.71)	5.21 (5.03, 5.39)	4.33 (4.17, 4.49)	**<0.0001**
	**Ref.**	**−10**	**−15**	**−29**	
Valine	4.14 (3.99, 4.30)	3.78 (3.63, 3.91)	3.60 (3.47, 3.73)	2.95 (2.85, 3.07)	**<0.0001**
	**Ref.**	**−9**	**−13**	**−29**	
					
*Other essential amino acids*					
Histidine	2.12 (2.04, 2.20)	1.83 (1.77, 1.90)	1.72 (1.66, 1.78)	1.52 (1.46, 1.57)	**<0.0001**
	**Ref.**	**−13**	**−19**	**−28**	
Lysine	5.01 (4.78, 5.24)	4.14 (3.97, 4.33)	3.76 (3.60, 3.93)	2.82 (2.69, 2.95)	**<0.0001**
	**Ref.**	**−17**	**−25**	**−44**	
Methionine	1.67 (1.60, 1.74)	1.38 (1.33, 1.43)	1.24 (1.20, 1.29)	0.88 (0.84, 0.92)	**<0.0001**
	**Ref.**	**−18**	**−26**	**−47**	
Phenylalanine	3.55 (3.43, 3.68)	3.34 (3.24, 3.46)	3.21 (3.11, 3.32)	2.93 (2.82, 3.03)	**<0.0001**
	**Ref.**	**−6**	**−10**	**−18**	
Threonine	2.99 (2.88, 3.10)	2.61 (2.52, 2.71)	2.43 (2.34, 2.52)	2.19 (2.11, 2.27)	**<0.0001**
	**Ref.**	**−13**	**−19**	**−27**	
Tryptophan	0.93 (0.90, 0.96)	0.86 (0.84, 0.89)	0.82 (0.79, 0.85)	0.77 (0.74, 0.79)	**<0.0001**
	**Ref.**	**−7**	**−12**	**−18**	

*Non-essential amino acids*					
Alanine	3.56 (3.42, 3.69)	2.97 (2.87, 3.08)	2.67 (2.57, 2.77)	2.63 (2.53, 2.73)	**<0.0001**
	**Ref.**	**−16**	**−25**	**−26**	
Arginine	4.13 (3.96, 4.31)	3.69 (3.54, 3.85)	3.36 (3.23, 3.51)	3.92 (3.75, 4.09)	**<0.0001**
	**Ref.**	**−11**	**−19**	**−5**	
Aspartate	7.01 (6.75, 7.28)	6.44 (6.21, 6.67)	6.00 (5.79, 6.22)	6.33 (6.10, 6.58)	**<0.0001**
	**Ref.**	**−8**	**−14**	**−10**	
Cystine	1.04 (1.00, 1.08)	0.94 (0.91, 0.97)	0.88 (0.85, 0.91)	0.84 (0.81, 0.87)	**<0.0001**
	**Ref.**	**−9**	**−15**	**−19**	
Glutamate	16.10 (15.59, 16.63)	15.56 (15.09, 16.05)	15.10 (14.64, 15.57)	14.06 (13.61, 14.52)	**<0.0001**
	**Ref.**	**−3**	**−6**	**−13**	
Glycine	3.12 (3.00, 3.25)	2.56 (2.46, 2.66)	2.28 (2.20, 2.37)	2.61 (2.50, 2.71)	**<0.0001**
	**Ref.**	**−18**	**−27**	**−16**	
Proline	5.61 (5.42, 5.80)	5.47 (5.30, 5.66)	5.47 (5.30, 5.66)	4.32 (4.17, 4.47)	**<0.0001**
	**Ref.**	**−2**	**−2**	**−23**	
Serine	3.70 (3.58, 3.84)	3.56 (3.44, 3.68)	3.43 (3.31, 3.54)	3.08 (2.97, 3.19)	**<0.0001**
	**Ref.**	**−4**	**−8**	**−17**	
Tyrosine	2.71 (2.61, 2.82)	2.48 (2.39, 2.57)	2.36 (2.28, 2.45)	1.86 (1.79, 1.94)	**<0.0001**
	**Ref.**	**−9**	**−13**	**−31**	

Abbreviation: Ref., reference.

a Adjusted for age (30–34; 35–39; 40–44; 45–49 years), body mass index (<22.5; 22.5–24.9; ⩾25 kg/m^2^; unknown), smoking status (never; former; current), alcohol intake (<1; 1–7; 8–15; ⩾16 g/d) and energy intake (continuously).

b *P*-values are for the difference across all four diet groups calculated by analysis of variance. Conventional *P*-values are shown, and those marked with in bold were significant after Bonferroni correction (*P*<0.0028).

**Table 4:** Spearman's rank correlations between plasma concentrations and dietary intakes of amino acids

	*Sample size*	r	P^a^
*Branched-chain essential amino acids*			
Isoleucine	392	0.10	0.05
Leucine	392	0.16	**0.001**
Valine	392	0.14	0.01
			
*Other essential amino acids*			
Histidine	392	−0.02	0.7
Lysine	381	0.21	**<0.0001**
Methionine	392	0.19	**0.0002**
Phenylalanine	299	0.05	0.4
Threonine	392	0.06	0.2
Tryptophan	390	0.16	**0.001**
			
*Non-essential amino acids*			
Alanine	392	−0.05	0.4
Arginine	392	0.09	0.07
Aspartate	392	0.004	0.9
Glutamate	392	0.03	0.6
Glycine	392	−0.15	0.003
Proline	347	0.12	0.02
Serine	392	0.04	0.4
Tyrosine	392	0.17	**0.0007**

a Conventional *P*-values are shown and those marked in bold were significant after Bonferroni correction (*P*<0.0029).
