# Supplementary material for: Plasma concentrations and intakes of amino acids in male meat-eaters, fish-eaters, vegetarians and vegans: a cross-sectional analysis in the EPIC-Oxford cohort
Source: Eur J Clin Nutr. 2015 Sep 23;70(3):306–12. doi: 10.1038/ejcn.2015.144 (PMC4705437; doi:10.1038/ejcn.2015.144)
Supplement: Supplementary Information [file ejcn2015144x1.doc]

**Supplementary Table 1. Factors related to blood collection and handling by habitual diet group**

|  | **Median (inter-quartile range) or n (%)^1^** | | | | **p_difference_^2^** |
| --- | --- | --- | --- | --- | --- |
|  | **Meat-eaters (n=98)** | **Fish-eaters (n=98)** | **Vegetarians (n=98)** | **Vegans (n=98)** |  |
| **Variables related to blood collection and handling** | |  |  |  |  |
| Postal recruitment | 61 (62.2) | 95 (96.9) | 97 (99.0) | 98 (100.0) | − |
| Medication or supplement at blood collection^3^, n (%) | 60 (61.2) | 61 (63.5) | 65 (66.3) | 66 (67.4) | 0.8 |
| Time since last food or drink at blood collection^3^, h | 2.0 (1.0, 3.5) | 2.3 (1.3, 4.5) | 2.4 (1.5, 4.5) | 2.5 (1.3, 4.3) | 0.03 |
| Time of blood collection^3^, h:min | 11:20 (10:00, 15:27) | 10:45 (09:28, 15:30) | 10:10 (09:30, 14:45) | 10:40 (09:45, 16:08) | 0.2 |
| Time from blood collection to processing^3^, h | 25.8 (23.4, 67.1) | 44.4 (26.1, 72.1) | 42.9 (25.0, 71.4) | 41.7 (24.7, 71.9) | 0.004 |
| Lipemic, n (%) | 23 (23.5) | 13 (13.3) | 24 (24.5) | 17 (17.4) | 0.2 |
| Hemolytic, n (%) | 1 (1.0) | 7 (7.1) | 7 (7.1) | 3 (3.1) | 0.8 |

^1^ Values are median (inter-quartile range) if not otherwise specified as n (%).

^2^ Differences between diet groups were tested using the Kruskal-Wallis ANOVA and χ^2^ test for continuous and categorical variables, respectively. However, no test was performed for recruitment method and Fisher's exact test was used for haemolytic, due to zero and low numbers, respectively, in some cells. Conventional p-values are shown and those marked in **bold** were significant after Bonferroni correction (p<0.0023; not all tests performed are shown).

^3^Unknown for some participants: medication or supplement at blood collection n=2; time since last food or drink at blood collection n=9; time of blood collection n=10; time from blood collection to processing n=10.

**Supplementary Table 2. Spearman's rank correlations between plasma amino acid concentrations and intake of protein (% of energy) from food types^1^.**

**A.** Plant products

|  | **Non-soya plant** | | **Soya** | |
| --- | --- | --- | --- | --- |
|  | **r** | **p^2^** | **r** | **p^2^** |
| **Branched-chain essential amino acids** | | | |  |
| Isoleucine | -0.10 | 0.04 | -0.13 | 0.01 |
| Leucine | -0.15 | 0.003 | -0.18 | 0.0004 |
| Valine | -0.14 | 0.005 | -0.19 | **0.0001** |
| **Other essential amino acids** | | | |  |
| Histidine | 0.01 | 0.8 | 0.02 | 0.7 |
| Lysine | -0.21 | **<0.0001** | -0.21 | **<0.0001** |
| Methionine | -0.13 | 0.008 | -0.19 | **0.0002** |
| Phenylalanine | -0.01 | 0.8 | -0.03 | 0.6 |
| Threonine | -0.02 | 0.7 | -0.03 | 0.5 |
| Tryptophan | -0.10 | 0.05 | -0.16 | 0.001 |
| **Non-essential amino acids** | | | |  |
| Alanine | 0.04 | 0.4 | 0.10 | 0.04 |
| Arginine | -0.06 | 0.3 | -0.11 | 0.04 |
| Asparagine | 0.08 | 0.1 | 0.07 | 0.2 |
| Aspartate | 0.06 | 0.2 | 0.03 | 0.6 |
| Glutamate | -0.06 | 0.2 | 0.02 | 0.7 |
| Glutamine | 0.09 | 0.07 | 0.07 | 0.2 |
| Glycine | 0.14 | 0.005 | 0.26 | **<0.0001** |
| Proline | 0.01 | 0.9 | -0.09 | 0.09 |
| Serine | 0.05 | 0.4 | 0.04 | 0.5 |
| Tyrosine | -0.19 | **0.0002** | -0.19 | **0.0002** |
| **Non-standard amino acids** | | | |  |
| Citrulline | 0.00 | 0.9 | 0.06 | 0.2 |
| Ornithine | 0.12 | 0.02 | 0.15 | 0.004 |

B. Animal products

|  | **Meat^4^** | | **Fish^5^** | | **Dairy^3^** | | **Eggs^3^** | | **Mixed animal^3^** | |
| --- | --- | --- | --- | --- | --- | --- | --- | --- | --- | --- |
|  | **r** | **p^2^** | **r** | **p^2^** | **r** | **p^2^** | **r** | **p^2^** | **r** | **p^2^** |
| **Branched-chain essential amino acids** | | | |  |  |  |  |  |  |  |
| Isoleucine | -0.03 | 0.8 | -0.13 | 0.07 | 0.18 | 0.002 | 0.06 | 0.3 | -0.01 | 0.9 |
| Leucine | -0.03 | 0.8 | -0.11 | 0.1 | 0.20 | 0.0006 | 0.09 | 0.1 | 0.01 | 0.9 |
| Valine | 0.13 | 0.2 | -0.06 | 0.4 | 0.15 | 0.01 | 0.18 | 0.002 | -0.02 | 0.8 |
| **Other essential amino acids** | | | |  |  |  |  |  |  |  |
| Histidine | -0.13 | 0.2 | -0.14 | 0.05 | -0.01 | 0.9 | 0.06 | 0.3 | -0.05 | 0.4 |
| Lysine | 0.06 | 0.5 | 0.00 | 1.0 | 0.15 | 0.01 | 0.16 | 0.008 | -0.06 | 0.3 |
| Methionine | 0.02 | 0.8 | -0.02 | 0.8 | 0.18 | 0.002 | 0.04 | 0.5 | -0.08 | 0.2 |
| Phenylalanine | -0.06 | 0.6 | -0.08 | 0.3 | -0.01 | 0.9 | 0.15 | 0.03 | -0.03 | 0.7 |
| Threonine | -0.04 | 0.7 | -0.04 | 0.6 | 0.06 | 0.3 | 0.12 | 0.04 | 0.06 | 0.3 |
| Tryptophan | -0.03 | 0.8 | 0.03 | 0.7 | 0.09 | 0.1 | 0.11 | 0.06 | -0.04 | 0.5 |
| **Non-essential amino acids** | | | |  |  |  |  |  |  |  |
| Alanine | -0.09 | 0.4 | -0.06 | 0.4 | 0.04 | 0.5 | 0.09 | 0.1 | -0.02 | 0.7 |
| Arginine | 0.05 | 0.6 | 0.04 | 0.6 | 0.04 | 0.5 | -0.12 | 0.03 | -0.10 | 0.1 |
| Asparagine | -0.15 | 0.1 | -0.03 | 0.7 | -0.02 | 0.8 | 0.11 | 0.07 | 0.08 | 0.1 |
| Aspartate | 0.03 | 0.8 | 0.10 | 0.1 | 0.10 | 0.08 | 0.14 | 0.02 | 0.04 | 0.5 |
| Glutamate | -0.12 | 0.2 | 0.02 | 0.8 | 0.05 | 0.4 | 0.20 | 0.0005 | 0.10 | 0.09 |
| Glutamine | 0.06 | 0.6 | -0.01 | 0.9 | -0.04 | 0.6 | -0.09 | 0.1 | -0.12 | 0.04 |
| Glycine | -0.15 | 0.1 | 0.01 | 0.9 | -0.02 | 0.8 | 0.18 | 0.003 | 0.09 | 0.1 |
| Proline | -0.04 | 0.7 | -0.20 | 0.01 | 0.03 | 0.6 | 0.08 | 0.2 | 0.05 | 0.4 |
| Serine | 0.05 | 0.6 | 0.03 | 0.7 | 0.06 | 0.3 | 0.05 | 0.4 | 0.06 | 0.3 |
| Tyrosine | 0.02 | 0.9 | -0.04 | 0.6 | 0.14 | 0.02 | 0.11 | 0.07 | -0.07 | 0.3 |
| **Non-standard amino acids** | | | |  |  |  |  |  |  |  |
| Citrulline | -0.08 | 0.4 | -0.08 | 0.3 | 0.16 | 0.005 | -0.14 | 0.02 | 0.04 | 0.5 |
| Ornithine | -0.15 | 0.1 | -0.13 | 0.07 | -0.04 | 0.4 | 0.14 | 0.02 | 0.01 | 0.9 |

^1^ Foods were categorised into food types based on the main protein source in the food.

^2^ Conventional p-values are shown and those marked in bold were significant after Bonferroni correction (p<0.00034).

^3^ Excluding vegans: n=294, except for lysine (n=285), phenylalanine (n=227), tryptophan (n=292), glutamine (n=293), proline (n=260) and ornithine (n=293).

^4^ In meat-eaters only: n=98, except for lysine (n=95), phenylalanine (n=78) and proline (n=89).

^5^ In meat-eaters and fish-eaters only: n=192, except for lysine (n=188), phenylalanine (n=147), tryptophan (n=191) and proline (n=170).
